# Maintaining Traditions: Food and Identity among Early Immigrants to Upper Canada

**DOI:** 10.1007/s41636-020-00237-5

**Published:** 2020-04-28

**Authors:** Eric D. Tourigny

**Affiliations:** School of History, Classics and Archaeology, Newcastle University, Newcastle upon Tyne, NE1 7RU UK

**Keywords:** foodways, immigrants, identity, zooarchaeology, Upper Canada

## Abstract

A critical examination of the relationships between food and identity is explored among early British and American Loyalist settlers in Upper Canada (southern Ontario) from the late 18th to the late 19th centuries. This research synthesizes zooarchaeological data from the region and interprets these alongside historical texts to address how meat was incorporated into early immigrant diets. Previous scholarship generally agreed that pork played a dominant role in Upper Canadian cuisine and that residents first settling in the area were particularly reliant on wild meat resources. Archaeological evidence suggests this was not the case. Results and discussions highlight the influence of British working-class traditions on Upper Canadian identities and the development of regional cuisines in southern Ontario. Parallels are drawn to anthropological and sociological studies of migrant foodways, encouraging archaeologists to consider the importance of maintaining food traditions when examining early immigrant assemblages.

## Extracto

Se examinan críticamente las relaciones entre la comida y la identidad entre los primeros colonos lealistas británicos y estadounidenses en el Alto Canadá (sur de Ontario) desde finales del siglo 18 hasta finales del 19. Esta investigación sintetiza datos zooarqueológicos de la región y los interpreta junto con textos históricos para abordar las formas en que la carne se incorporó a las primeras dietas de los inmigrantes. Los estudios anteriores generalmente coincidieron en que la carne de cerdo desempeñaba un papel dominante en la cocina del Alto Canadá y que los residentes que se establecieron por primera vez en el área dependían particularmente de los recursos de carne silvestre. La evidencia arqueológica sugiere que este no fue el caso. Los resultados y las discusiones resaltan la influencia de las tradiciones de la clase trabajadora británica en las identidades del Alto Canadá y el desarrollo de las cocinas regionales en el sur de Ontario. Se trazan aspectos paralelos con los estudios antropológicos y sociológicos de las vías alimentarias de los migrantes, alentando a los arqueólogos a considerar la importancia del mantenimiento de las tradiciones alimentarias en el examen de los primeros grupos de inmigrantes.

## Résumé

Les relations entre les aliments et l'identité parmi les premiers pionniers loyalistes britanniques et américains dans le Haut-Canada (Ontario du Sud) à compter de la fin du 18ème siècle jusqu'à la fin du 19ème siècle font l'objet d'un examen critique. Cette recherche effectue une synthèse de données zooarchéologiques collectées dans la région pour ensuite les interpréter conjointement à des textes historiques afin d'étudier les voies par lesquelles la viande a été introduite dans les régimes alimentaires des premiers immigrants. La recherche antérieure s'accordait généralement sur le fait que le porc jouait un rôle dominant dans la cuisine du Haut-Canada, et que les résidents qui se sont initialement installés dans la région s'appuyaient particulièrement sur des ressources de gibier. Les preuves archéologiques suggèrent que tel n'était pas le cas. Les résultats et les discussions mettent en lumière l'influence des traditions de la classe ouvrière britannique sur les identités du Haut-Canada et le développement des cuisines régionales dans l'Ontario du Sud. Des parallèles sont établis avec les études anthropologiques et sociologiques des régimes alimentaires, incitant les archéologues à prendre en compte l'importance de la préservation des traditions culinaires dans l'étude des assemblages des premiers immigrants.

## Introduction

The foods people eat can be reflective of and used toward an active negotiation of identity (A. Jones and Richards 2003; O’Sullivan 2003; Sykes 2004; K. Lewis 2007; Twiss 2007, 2012). Over the past few decades, archaeological studies linking food and social diversity have become increasingly popular in North American historical archaeology (Landon 1987b; Scott 1996; Warner 1998; Franklin 2001; Milne and Crabtree 2001; Groover and Homes Hogue 2014; Dappert-Coonrod and Kuehn 2017). Foodways research does not simply look at what people ate, but represents a critical examination of the ways people thought about and interacted with food: how and why they obtained it, distributed it, prepared it, preserved it, and consumed it (Anderson 1971:29). The usefulness of this concept as an interpretive framework lies in its all-encompassing definition as an interrelated network of decisions affecting the ways individuals eat. It helps archaeologists move beyond simply listing the foods people ate by recognizing that dietary components and food behaviors are influenced by a series of complex factors related to social diversity (ethnicity, gender, religious belief, socioeconomic status), thus contributing to an individual’s and/or group’s sense of identity (Landon 1996:3, 2002:247; Kuehn 2007:200; Twiss 2012:381).

Despite many zooarchaeological studies of the historical period published in the United States (for a summary, see Landon [2009]) and a growing body of work from Quebec and the Maritime provinces (Cossette 2000; Cossette and Horard-Herbin 2003; Hodgetts 2006; Bernard 2012; Tourigny and Noël 2013; Betts et al. 2014), few publications, apart from a handful of case studies, describe faunal evidence from European Canadian assemblages in Ontario (Ferris and I. Kenyon 1983; Betts 2000; MacDonald and Needs-Howarth 2013). Most faunal analyses in Ontario are produced for the commercial sector, and, while many qualified zooarchaeologists are regularly analyzing and reporting on historical assemblages, their reports remain hidden in the gray literature (Tourigny 2017b). The zooarchaeologists working in the province may have a good idea as to what they expect to find in historical period assemblages; however, time and budget constraints rarely afford them the opportunity to conduct proper comparisons or address broader research questions aimed at synthesizing trends visible across a wider region or time period.

This article collates zooarchaeological data from early immigrant and immigrant-descendent contexts excavated from across southern and eastern Upper Canada. Assemblages consist of household deposits associated with residents of British or American Loyalist heritage. Through a comparison of what people said they were eating with the physical evidence of the food remains, this article explores the ways local foodways developed and came to represent early expressions of identity in this part of the world. Did British and American immigrants (and their descendants) make efforts to maintain foodways that were traditional to them? Or, did they develop new food traditions upon settling in the province? Focusing on meat-consumption habits, faunal data are qualified and quantified in both time and space to highlight trends in consumption practices. Comparisons are then made with contemporaneous foodways derived from data gathered elsewhere in North America and in the United Kingdom.

## Upper Canada and Its Food History

**Fig. 1:**
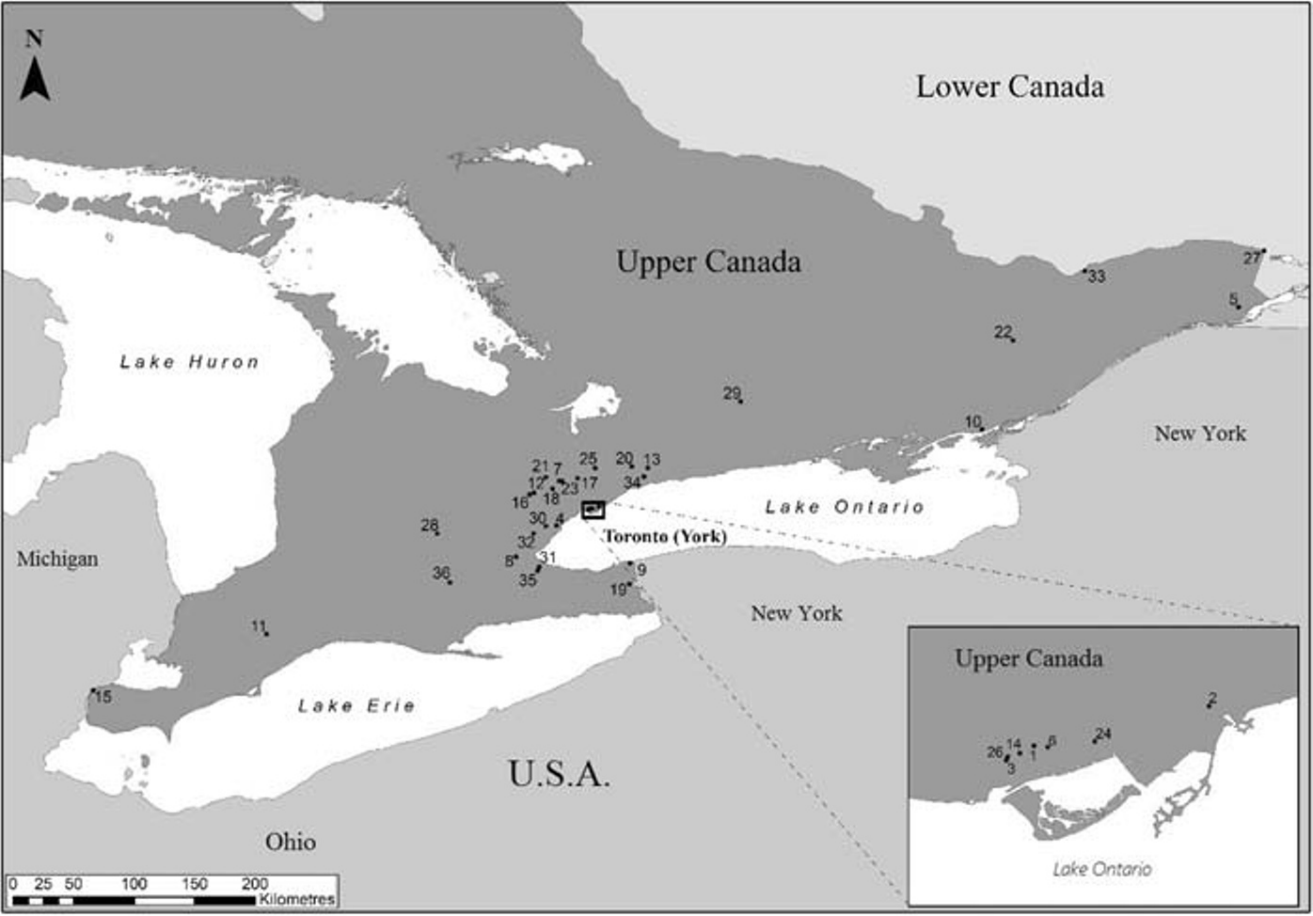
Locations of sites within Upper Canada. Numbers refer to sites listed in Table 1. (Figure by Nicky Garland, 2018.)

Established in 1791, the province of Upper Canada stretched along the northern shores of the Great Lakes, comprising what is now the southern part of the province of Ontario (Fig. 1). It was initially formed, in part, to accommodate United Empire Loyalists as refugees from the American Revolution (Taylor 2007). Prior to its establishment, few people of European ancestry inhabited the area, which was claimed by the French until 1763. Taking over these claims from France, the British government created an English-speaking jurisdiction, separating Upper Canada (English) from Lower Canada (mostly the French-speaking, modern province of Quebec). An initial influx of Loyalist settlers arrived in Upper Canada between 1783 and 1789, following the end of the American Revolutionary War (Taylor 2007). Immigrants were encouraged to settle in the Canadas, where they were offered free (or very affordable) parcels of land, the necessary equipment to establish new farms, as well as two years of food rations to help them through the initial settling period (R. Jones 1946:17; Russell 1973:15–16). Increased migration from the United States later led to a second wave of “late Loyalists” between 1792 and 1812, which saw Upper Canada’s population grow from 14,000 in 1791 to 70,000 by 1811 (Taylor 2007:19). Concurrently, exploding urban populations and high levels of unemployment in Britain led to the appearance of government-, private-, and parish-sponsored relocation programs that would see nearly a million settlers relocate to British North America from 1815 through to the 1830s (Russell 1973:18; Karr 1974; Coleman 1978; Flanders 2004:xxxvi; Lee 2004). The Irish Potato Famine and the disposition of Scottish Crofters also resulted in a large movement of people to Upper Canada. The province was the fastest growing North American region between 1825 and 1851 (Russell 1973:10; Careless 1984:79; F. Lewis 2001:175).

The current understanding of 19th-century Upper Canadian foodways is primarily informed by a series of published historical accounts interpreted alongside knowledge of 18th- and 19th-century British and American customs (U. Abrahamson 1967; Boyce 1972; H. Abrahamson 1981; I. Kenyon and S. Kenyon 1992). These studies suggest Upper Canadian meals were slightly different from those in prevailing English customs of the time, with a focus on dietary staples, such as meats, breads, and tea (Traill 1846, 1857; Moodie 1852, 1853). Local wild ingredients, such as maple sugar, maize, pumpkin, and a variety of wild fruits, were reportedly common at the Upper Canadian table (Abonyi 1993), along with various types of wild meat, including venison, turkey, partridge, passenger pigeon, squirrel, hare, duck, and other fowl (Traill 1846, 1857; Moodie 1852, 1853; Robertson 1911:131; Greenberg 2014). Documents suggest those living in more rural areas had better access to wild game, either as a result of increased availability through hunting or through advantageous trading opportunities with indigenous peoples (Traill 1857:155; Langton 1926:35; Radcliffe 1952; Cameron et al. 2000:23,65,85,115,170–173,180; Fairburn 2013:68). Few of these documents mention fish as a staple food item, despite their ready availability in the many freshwater lakes and rivers.

Upper Canadian meals were described as containing noticeably more meat than typical English menus, but were criticized for a lack of variety in the ways they were prepared and served (I. Kenyon and S. Kenyon 1992:8). Dry (but greasy) dishes that were particularly reliant on pork and potatoes were considered characteristic of the region (Bates 1978; I. Kenyon and S. Kenyon 1992:9). Urban centers held markets where domestic and wild meats were available for purchase (I. Kenyon and S. Kenyon 1992:5), and meat products were available in both fresh and preserved forms, with salted pork particularly being linked to Upper Canadian diets (Tourigny 2017a). Residents were thought to rely on breads, porridges, cornmeal, and wild foods at times of low supply (Boyce 1972; I. Kenyon and S. Kenyon 1992). Rural areas were thought to be reliant on pork, especially amongst newly settled immigrant families (Moodie 1852:357; I. Kenyon and S. Kenyon 1992; James 1997:28). Pigs could be fed on almost anything, fattened quite readily, and were easy to care for, therefore quickly supplying new settlers with a source of fresh meat. The ability to rear cattle and sheep is thought to have improved as farmers became better established; as more land was cleared for pasture and enclosures were built to keep away predators (Need 1838:90; Ferris and I. Kenyon 1983). Food was not always abundant, and rural residents often required access to markets for the resources they could not produce themselves (Traill 1857:124). Many farmers living on the frontier looked to participate in the market economy, but long distances and lack of time and resources did not always allow it (M’Donald 1822; Smith 1923; Henretta 1978; James 1997). The late 19th century is generally seen as one marked by a refinement in food preparation and manufacturing. Infrastructure and technology dramatically improved life for those living in formerly remote, rural settlements as travel to and from city markets became easier (Bates 1978:44–45).

Previous archaeological research on Upper Canadian foodways has come from investigations of human skeletal, paleobotanical, and zooarchaeological remains. Studies of human dental pathologies and stable isotopes have identified diets rich in carbohydrates and sugars as compared to those associated with British and American skeletal assemblages (Saunders et al. 1997; Blackbourn 2005). The analysis of botanical remains at a 19th-century fort in Prescott, Ontario, identified mostly locally grown plants with a few imported species (Moyle 1994; Lyall 2010), thus supporting the idea that Upper Canadians included indigenous flora in their diets. Most academic studies of animal bones relate to excavations undertaken by Parks Canada (Rick 1993; Betts 2000) and a few dissertations and publications (Ferris and I. Kenyon 1983; Henderson 1992; James 1997; Beaudoin 2013; MacDonald and Needs-Howarth 2013). The former inform on the provisioning of military sites, while the latter provide summaries and interpretations of one or a handful of rural domestic sites occupied between the late 18th and early 20th centuries. Based on observations of five different assemblages, Ferris and I. Kenyon (1983) noted Scottish immigrants consumed more mutton, while James’s (1997) investigation of butchery practices noted that roasts were generally preferred in Upper Canada. While slight variation in butchery practices is observed, the evidence suggests most followed an increasingly standardized carcass-division technique witnessed throughout English-speaking North America (James 1997; Tourigny 2017a).

## Late Eighteenth- and Early Nineteenth-Century British and American Foodways

Late 18th- and early 19th-century foodways in Britain and colonial British North America were highly influenced by tradition, the history of colonialism, and industrial and technological developments (Rixson 2000; Broomfield 2007). Developing technologies permitted new ways to create, distribute, preserve, consume, and appreciate food; however, their availability did not always equate to a rapid adoption (Rixson 2000). Prior to the urban boom of the late 18th century, it was common for households to raise livestock (especially pigs and poultry) and maintain kitchen gardens or allotments to grow their own produce. Communal lands on which anyone could raise livestock and grow crops were occasionally available, although rapidly disappearing (Broomfield 2007:4; Tarlow 2007:42–50). People ventured to the nearest market town for the items they could not produce themselves (Broomfield 2007:4). Access to gardens, livestock and common lands meant that even those of lower socioeconomic standing could have a varied diet, including protein and vegetables. Common vegetables included lettuce, cucumbers, radishes, peas, and a variety of root vegetables that could be stored for winter consumption. Cabbages and kale were popular winter vegetables (Broomfield 2007:4–5). Staple grains included oats, wheat, and barley. Dairy products, such as milk and butter, were easily accessible through neighboring farmers. Land-management changes in the 17th century led to reduced access to common lands, while the Industrial Revolution and a rise in urban populations further affected access to and availability of foodstuffs for people of differing socioeconomic classes (Broomfield 2007:5; Tarlow 2007:42–50).

By the late 18th and early 19th centuries, perishable foods could be preserved by drying, smoking, pickling, salting, or stewing in sugar (Broomfield 2007:5; Tourigny 2017a). Summer provided difficult conditions for keeping meat; however, most animals were then too lean to slaughter. It was not until the late fall or early winter, once animals had fattened off the stubble of the fall harvest, that they were ready for slaughter. The colder weather helped with the preservation of their carcasses (Broomfield 2007:3), and meats not immediately consumed could be preserved for later use. Foodways were, nonetheless, marked by the seasonality of available foods. By the late 18th century, fruits and vegetables could be preserved in canning jars, and it was not until the second decade of the 19th century that tin cans were used (Spencer 2004:282). As the century progressed, new technologies emerged allowing perishable items to keep longer and travel farther. Steamships and steam-powered railway cars moved products for greater distances in shortened times. Increased speed and efficiency also led to lower market prices (Broomfield 2007:19). The importance of cooler temperatures was well known, and by the mid-19th century insulated transport holds packed with ice were used to carry chilled carcasses (Rixson 2000; Reynolds et al. 2014). Ice huts or icehouses were employed in some of the wealthier British homes in the later postmedieval period as a way to keep food cold during the summer. A mechanical cold-air system was first used in 1879 to transport frozen shipments of meat (Rixson 2000:274), but household refrigeration units only became common in the 20th century (Hempstead and Worthington 2004:673).

In the academic literature, dietary practices and local attitudes toward food in colonial North America are described from both historical and archaeological perspectives. The majority of zooarchaeological studies in British North America have focused on the Chesapeake and New England areas during the 17th and 18th centuries (Bowen 1975, 1990, 1992, 1998; Miller 1984, 1988; Scott 1985, 1991, 1996; Singer 1985; Landon 1987a, 1987b, 1993, 1996, 1997; Bowen and Manning 1994; Walsh et al. 1997; Bowen and Trevarthen-Andrews 2000; Milne and Crabtree 2001). Many studies also describe foodways related to 18th- and 19th-century western expansion (Dixon 2005; Kuehn 2007; Reynolds et al. 2014). Many of the settlers arriving in Upper Canada in the late 18th and early 19th centuries were Americans choosing to remain loyal to the British Crown following the United States’ newly won independence; therefore, it is important to consider the dietary habits that formed earlier in the United States. Miller’s (1984, 1988) analyses of 17th- and 18th-century sites in the Chesapeake Bay area provide some understanding of earlier British-colonial food-consumption habits. He notes that wild animals, including deer, small mammals, fish, and wildfowl, played a more prominent role in earlier colonial diets, where the most important domesticates consisted of cattle and pigs. Sheep, he says, did not maintain as important a role in the Chesapeake as in Britain. Seasonal variability in diet was highly marked, as people depended on the differential availability of wild and farmed resources. Domestic animals were relied upon in the fall and early winter, and wild resources were exploited in the spring and summer months (Shapiro 1979; Miller 1984, 1988). Such seasonal slaughtering cycles were observed in other parts of the United States and extended to both urban and rural areas (Bowen 1988; Landon 1991, 1993, 2008). By the second half of the 17th century, general dietary patterns based on beef and pork consumption became increasingly uniform, and dependence on wild resources significantly decreased. The loss of self-sufficiency by urban dwellers, first described in Britain, was also observed in America (Pendery 1984:22; Landon 1996; Walsh et al. 1997).

A similar pattern is identified in early 19th-century settlements in the Midwest, whereby residents first relied on wild resources and depended increasingly on beef and pork as the century progressed (Groover and Homes Hogue 2014). By the second half of the 19th century, an increased availability of preserved and processed foods, combined with the improved transportation of commercial products, resulted in a greater consumption of meat imported from distant locations and a further reduction of the incorporation of wild taxa on sites in the northeastern United States (Skags 1986; Rixson 2000; Reynolds et al. 2014). Kuehn (2007:203) suggests a combination of the increased availability of imported foodstuffs along with resource depletion as a reason for the homogenization of diets throughout the Northeast and in northern Illinois and southern Wisconsin. Analyses of a late 18th-century British fort located in a remote location (Fort Michilimackinac) indicate British diet at the time was heavily reliant on meat from domestic animals, but included slightly more wild animals relative to British sites farther east (Scott 1996). In 19th-century Britain, diets were generally based on the consumption of domesticates (beef, pork, mutton, and chicken), while venison only became available to the general populace in 1831 (Mayhew 1967:120). Unfortunately, few zooarchaeological studies of 19th-century British assemblages are available to inform on what the archaeological signatures of these diets look like (Thomas 2009; Walczesky 2013:21; Gordon 2015). Gordon’s (2015) investigation of urban animal-bone assemblages in postmedieval Britain identifies a general increase in the consumption of cattle, and she relates this to the rise of the dairying industry. She also notes regional variations related to specializations in other livestock throughout different parts of the country and a slight increase in the consumption of wild animals linked to a rise in the urban upper-middle class and improved market provisioning. In Lower Canada, a few studies comparing late 18th- and early 19th-century French and British assemblages note the French incorporated more birds, pork, and wild game into their diets as opposed to the British, who consumed more beef and had a less varied diet overall (Coté 2005; Bernard 2012; Walczesky 2013).

## Materials and Methods

Zooarchaeological data were assembled from 36 sites located throughout southern and eastern Ontario (Fig. 1) (Table 1). These were excavated over the past 35 years by commercial archaeology units or state-run heritage-management organizations. Most of these assemblages were collected from soils sieved through 6 mm mesh, as has been the standard in Ontario since 2011 (Ministry of Tourism, Sport and Culture 2011). Assemblages recovered prior to 2011 likely employed 6 mm mesh, as was the generally accepted standard in the province and is usually indicated in the reports.

**Table 1:** List of assemblages

Site	Borden Number	Date (approximate)	Site Type	Source
1. Queen Street West (3 deposits: F36, F38, F46)	AjGu-63	1830s–1850s	Urban	Tourigny 2016
2. Ashbridge Estate (2 phases)	AjGt-1	1796–1913, 1904–1970	Rural	Tourigny 2016
3. Bell	AjGu-68	1840–1870s	Urban	Tourigny 2016
4. Benares	AjGv-30	1835–1857	Rural	James 1997
5. Bethune-Thompson House	BgFp-39	1873–1905	Rural	Casey 1994
6. Bishop's Block (4 houses)	AjGu-49	Late 19th–early 20th cent.	Urban	Needs-Howarth 2011b; Tourigny 2016
7. Block 55 H3	AlGv-383	1831–1860s	Rural	Berg 2015
8. Botanical Views Estate	AhGx-273	19th cent.	Rural	Prevec 1992
9. Butler	AhGs-18	1784–1813	Rural	Needs-Howarth 2009a
10. Cartwright Compound (3 phases)	BbGc-92	Late 18th cent., early 19th cent., early to mid-19th cent.	Urban	Needs-Howarth 2009b
11. Crinan Creek	AdHj-15	1850–1860	Rural	Prevec 1982
12. Deacon	AkGw-428	1828–1850s	Rural	Needs-Howarth 2014b
13. Delong 1	AlGr-139	1830–1870	Rural	Berg 2014a
14. Dollery (2 houses)	AjGu-81	1855–1878	Urban	Needs-Howarth 2012; Tourigny 2016
15. Duff-Bâby	AbHs-10	1798–1850	Urban	James 1997
16. Dunsmore	AkGw-397	1840s–1900	Rural	Berg 2014b
17. Edgar	AlGu-196	1830s–1870	Rural	Needs-Howarth 2006
18. Fletcher	AkGv-74	1840–1860	Rural	Prevec 1989
19. Fralick's Tavern	?	1840s–1850s	Urban	Prevec 2001
20. Graham	AlGs-370	1830s–late 19th cent.	Rural	Tourigny 2016
21. Hall	AlGw-68	1850s–1910s	Rural	Tourigny 2016
22. Inge-va	BfGb-2	1823–late 19th cent.	Urban	Dieterman 1988
23. John Beaton II	AlGv-219	1840s–1870s	Rural	Tourigny 2016
24. King-Caroline	AjGu-82	1820–1870	Urban	Needs-Howarth 2013
25. Lewis (2 phases)	AlGu-365	1825–1850, 1870–1880	Rural	Tourigny 2016
26. Lowry-Hannon	AjGu-79	Mid- to late 19th cent.	Urban	Needs-Howarth 2014c
27. Macdonell	BjFo-2	1788–1850	Rural	James 1997
28. Marsden	AiHd-105	Mid-19th cent.	Urban	Prevec 1995
29. Moodie	BcGn-9	1833–1860s	Rural	James 1997
30. Rasputine	AjGw-34	1900s	Rural	Prevec 1983
31. Smith's Knoll	AhGw-132	1875–1910	Urban	Prevec 1998, 1999
32. Speers	AiGw-547	1830s–1860s	Rural	Needs-Howarth 2014a
33. Ste. Famille School	BiFw-88	1861–1881	Urban	Needs-Howarth 2009b
34. Wilson Tenant	AlGu-51	1830s–1850s	Rural	Berg 2014c
35. Yeager	AhGw-256	1830s–1850s	Rural	Needs-Howarth 2011a
36. Yeigh	AgHc-1	1803–1829	Rural	Prevec 1981

Seven assemblages were identified and analyzed by the author (Tourigny 2016). Identifications were made to as precise a taxonomic level as possible, based on morphology and comparisons to reference materials in the Howard G. Savage Faunal Collection at the University of Toronto. Information was recorded on species present, body-portion representation, butchery, taphonomy, and pathologies. Age at death was estimated according to the state of epiphyseal fusion and tooth eruption and wear following criteria set out in relevant publications (Silver 1969; Chaplin 1971; Maltby 1979; Bull and Payne 1982; Grant 1982; Payne 1984; Legge 1992, 2013). These archaeological assemblages were from the Toronto region, which served as a case study for a Ph.D. dissertation that was later expanded to include other assemblages from across the province (Tourigny 2016). Those additional data were collected by other zooarchaeologists and gathered from reports in the gray literature (Table 1). Data-collection standards differ widely between zooarchaeologists due to the lack of clarity in the provincial standards and guidelines (Ministry of Tourism, Sport and Culture 2011; Tourigny 2017b); however, reporting the number of identified specimens (NISP) is consistent and comparable among researchers. Reports used in this research were selected based on the completeness of the data, the integrity of the archaeological context, and the reputation of the zooarchaeologist.

Every effort was taken to separate sheep (*Ovis aries*) from goat (*Capra hircus*) specimens; however, caprine skeletal morphology makes it difficult to distinguish between them, and most analysts simply end up recording these specimens as “caprines.” While goats were present in the region, the majority of historical documents discuss sheep. When local zooarchaeologists are able to distinguish between species, most of those identifications are of sheep. Therefore, the following discussion makes the assumption that the majority of caprine specimens in the province represent sheep, as is standard practice when considering faunal assemblages in southern Ontario and historical northeastern North America (Bowen 1975; James 1997; Archaeological Services, Inc. 2007; Tourigny 2016, 2017b).

## Results

**Fig. 2:**
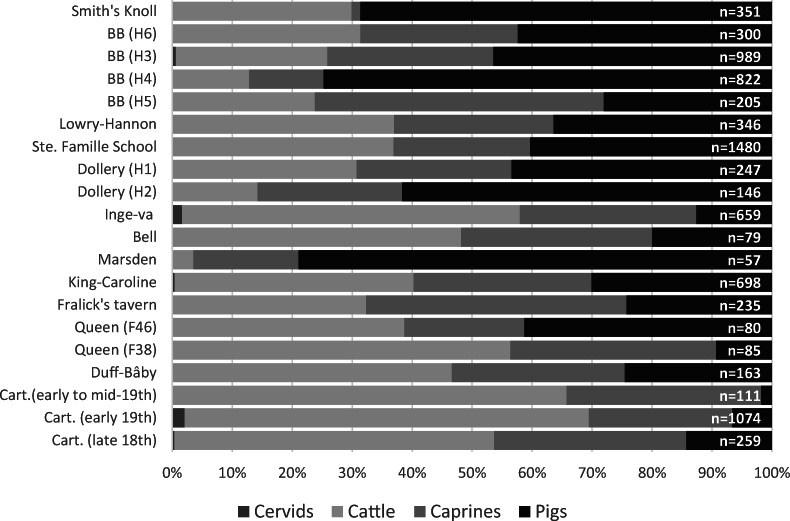
Distribution of artiodactyl specimens within urban assemblages according to percentage of NISP. Assemblages are presented by median date, with those dating to the late 18th/early 19th centuries at the bottom and assemblages dating to the late 19th century toward the top. (Figure by author, 2018.)

**Fig 3:**
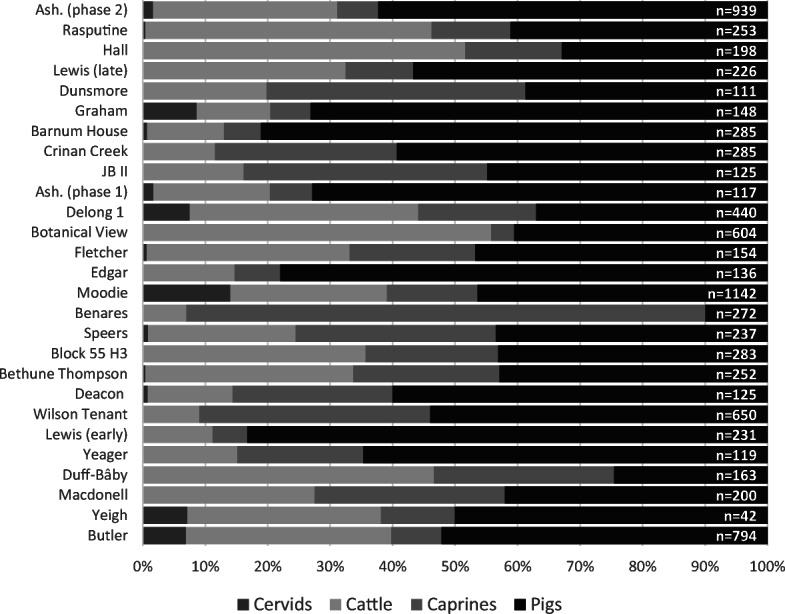
Distribution of artiodactyl specimens within rural assemblages according to percentage of NISP. Assemblages are presented by median date, with those dating to the late 18th/early 19th centuries at the bottom and assemblages dating to the late 19th century toward the top. (Figure by author, 2018.)

Faunal assemblages from this time period are mostly dominated by three mammalian species: cattle (*Bos taurus*), pigs (*Sus scrofa*), and sheep (*Ovis aries*) (Tourigny 2016). Figures 2 and 3 compare the proportion of artiodactyl specimens identified at each site. Results are variable in terms of which species is most commonly identified within an assemblage, but the majority of sites are dominated by either cattle or pigs, while only a few sites (e.g., House 5 at Bishop’s Block, Dunsmore, and Benares) have more sheep. Pigs are more dominant in rural assemblages, while cattle are more likely to form the largest component of earlier urban assemblages, as pig remains become more prominent among late 19th-century sites. The proportion of sheep remains is variable between assemblages, and the evidence suggests most of these were consumed as mutton (Tourigny 2016:239).

The majority of Cervidae identifications represent white-tailed deer (*Odocoileus virginianus*), while a handful of specimens represent moose (*Alces alces*) or elk (*Cervus canadensis*). The lack of moose and elk is unsurprising, as they are not common in the Carolinian forests that dominate most of the study region; however, the overall lack of deer is noteworthy and forms an important point of discussion in the following section. Cervids form a very small proportion of only a few assemblages and are only slightly more common among rural sites (48% of rural assemblages contain at least one specimen). Only four urban assemblages contain evidence of cervids, and there is no evidence to suggest these are more prevalent among early assemblages.

Age-at-death data are summarized elsewhere (Tourigny 2016, 2017a) and suggest cattle and pigs were primarily raised for meat, with the majority slaughtered soon after reaching peak weight. A few individuals lived longer to serve as breeding stock. Butchery marks, body-portion representation, and stable-isotope analyses suggest much of the pork consumed in Toronto throughout the 19th century came in the form of barreled meat. Some pork barrels were imported from the United States, while only locally raised beef was consumed (Guiry et al. 2017; Tourigny 2017a).

**Fig. 4:**
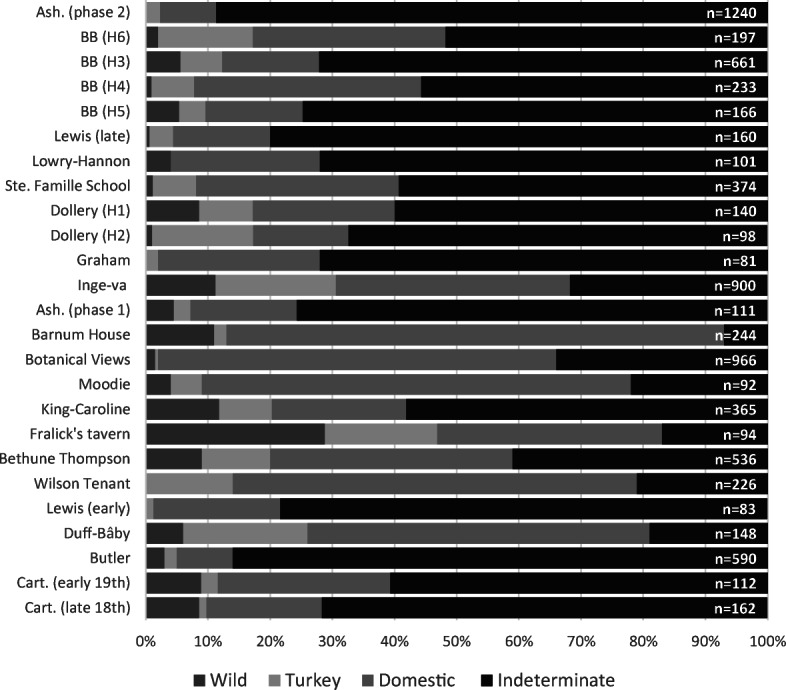
Distribution of bird specimens according to percentage of NISP. Assemblages are presented by median date, with those dating to the late 18th/early 19th centuries at the bottom and assemblages dating to the late 19th century toward the top. (Figure by author, 2018.)

While there is a notable absence of wild mammals contributing to early European Canadian diets, the same cannot be said of wild fowl and freshwater fish. Figure 4 makes evident the presence of wild fowl (mostly local duck species) at both rural and urban sites. Domestic birds, such as chickens (*Gallus gallus*) and greylag geese (*Anser anser*), form the highest proportion of identified bird specimens. Perhaps unexpectedly, urban sites appear to have larger proportions of wild bird specimens. Wild and domestic turkey represent the same species (*Meleagris gallopavo*), and their skeletons cannot be distinguished by gross morphological comparisons. Wild turkeys were and continue to be present in southern Ontario, and, therefore, remains could be representative of either. The majority of turkeys consumed in urban Toronto at that time were likely farm-raised, since a quarter to half of the turkey bones recovered from its sites are of skeletally immature individuals (Tourigny 2016:144), suggesting turkeys were husbanded for meat (Fothergill 2012). A slight decrease in identified wild fowl is seen amongst later assemblages.

**Fig. 5:**
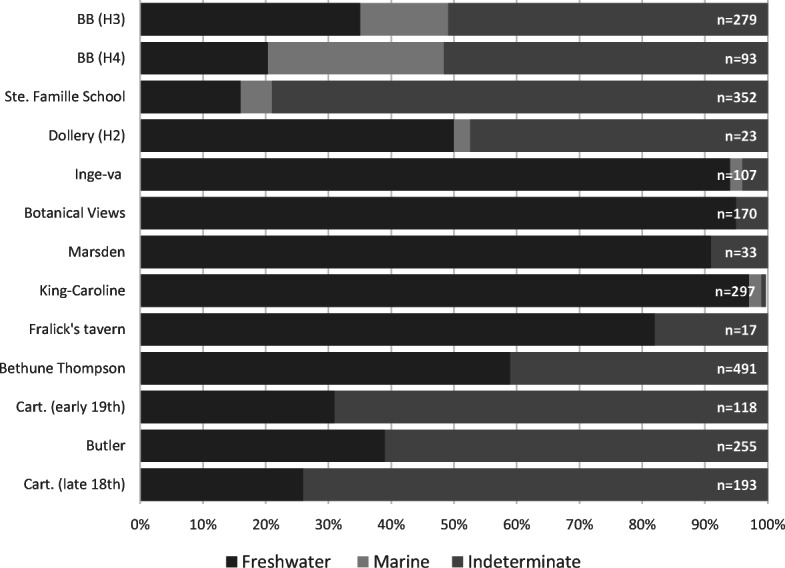
Distribution of fish specimens according to percentage of NISP. Assemblages are presented by median date, with those dating to the late 18th/early 19th centuries at the bottom and assemblages dating to the late 19th century toward the top. (Figure by author, 2018.)

Identifying the role of fish in historical European Canadian diets based on the recovery of faunal remains is complicated by the fact that few fish remains are recovered archaeologically in the province. Standard excavation strategies in Ontario employ screen sizes that are biased against the recovery of small-vertebrate remains (Ministry of Tourism, Sport and Culture 2011; Hawkins 2017:324; Tourigny 2017b). Figure 5 identifies the study sites with the most fish remains and breaks these down according to freshwater, marine, or indeterminate identifications. The majority of specimens identifiable to species level are of locally available freshwater fish. Marine fish are only identified amongst urban deposits and only outnumber freshwater fish identifications in one instance (House 4 deposit at Bishop’s Block). The “indeterminate” category includes unidentifiable remains and Atlantic salmon (*Salmo salar*), which were present in the Lake Ontario drainage basin (Parsons 1973; Dunfield 1985). Lake Ontario salmon populations were not anadromous (Guiry et al. 2016), but their remains cannot be visually distinguished from marine salmon of the same species that were imported. Older reports generally contained few fish identified to species, as it was once standard practice in Ontario zooarchaeology to exclude fish vertebrae from identifications (Hawkins 2017:316).

## Plenty of Game? American and British Working-Class Influences on Upper Canadian Foodways

Multiple accounts from early settlers discuss the importance and abundance of wild fowl and game (especially venison) upon arrival to the province. The following excerpts from letters, books, and personal correspondence highlight this:

They who live in the backwoods often have venison brought in either by their own people or by the Indian hunters who gladly exchange it for salt-pork, flour or vegetables. (Traill 1857:151)

For game—we have an abundance of venison, which is becoming more plentiful as the clearings increase affording them more food and driving off the wolves; you may buy it [venison] off the Indians at [one pence half-penny per pound], and sometimes for less [John Langton of Peterborough writing to his father in England on 31 October 1833]. (Langton 1926:35)

We have plenty of game here, and deer plenty, your gun has killed three deer, we have all liberty to carry a gun [William Voice of Blandford (Woodstock) writing to his sister in West Sussex on 27 October 1834]. (Cameron et al. 2000:170–173)

There is plenty of deer, rabbits, pheasants and pigeons to shoot at [Edward and Catherine Boxall of Adelaide Township writing to “Mother” in England on 28 July 1832]. (Cameron et al. 2000:85)

We have plenty of deer, rabbits, black squirrels, racoons, porcupines, ground hogs that are all good for food. ... The Indians ... love hunting. They will bring venison cheaper than we can kill it [William Cooper of Adelaide Township writing to his brother in West Sussex on 5 February 1833]. (Cameron et al. 2000:98)

And we have plenty of game in America; plenty of deer, turkeys, pheasants, partridges, and black squirrels and red squirrels [William Pannell of London District writing to his father and mother in West Sussex on 14 October 1832]. (Cameron et al. 2000:65)

We take our gun and go deer hunting when we want [James S. and William Goldring of York writing to their uncle in West Sussex on 9 April 1833]. (Cameron et al. 2000:115)

At times, I have seen fine deer pass close by my house, but they took great care not to wait until I had got my gun out for them; not but we get a great plenty of venison at [one pence half-penny] per pound [John and Ann Gamblen of Blandford (Woodstock) writing to Daniel King in Brighton on 18 February 1835]. (Cameron et al. 2000:180)

The importance attributed to venison and other wild game in historical documents makes it surprising to observe such a dearth of wild fauna in the archaeological record. Contradicting popular historical narratives, there is no evidence to suggest earlier settlements were more reliant on venison than later ones, or that rural settlements needed to rely on wild game for sustenance. The evidence suggests rural residents had access to and relied mostly on beef, pork, and mutton without having to wait until later in the 19th century for improved access to market towns.

These results differ from archaeological deposits related to the initial British settlement of the United States, where venison played an important role in local diets until settlers became better established. Scholarship suggests venison was no longer an important part of northeastern American foodways by the 18th century (Miller 1984, 1988; Pendery 1984; Walsh et al. 1997; Landon 2009), and most contemporary American archaeological assemblages also feature few wild mammal remains (Pendery 1984; Kuehn 2007; Landon 2009:88). There is a possibility that the lack of venison may be the result of the extirpation of local deer populations, as forests became increasingly sparse and wild fauna became marginalized. If this was the case, one would still expect deer to be prevalent in rural areas, where new expanses of agricultural land actually improved and expanded white-tailed deer habitats (Waller and Alverson 1997:217). Elsewhere, some have considered the possibility of overhunting leading to collapses in the deer population (Kuehn 2007). If this was the case in Ontario, one would expect earlier assemblages to contain higher proportions of deer.

Unlike Loyalist settlers, British immigrants arriving in Upper Canada in the late 18th and early 19th centuries would have found themselves in an environment very different from that to which they were accustomed. In order to explain their rejection of wild meat, it cannot be automatically concluded that wild Canadian fauna would have been “new” to British foodways. Many similar species existed in Britain, and there is no evidence to suggest British immigrants would have related to Canadian species any differently: white-tailed deer in Canada could occupy the same role as red deer (*Cervus elaphus*) in Britain, and grouse in Canada the same role as gray partridge (*Perdix perdix*) in Britain. Personal correspondence, like the passages presented above, makes simple references to categories of animals like deer without explanation of differences from British taxa. This familiarity with North American species by virtue of having similar taxa in Britain meant British immigrants could easily fit wild Canadian taxa into preexisting foodways.

Venison never formed a part of traditional British foodways, at least, not for most of its citizens. Since the medieval period, deer hunts were considered a noble activity, and the law ensured that consuming venison was reserved for the elite. Although members of the emergent middle classes could consume venison after laws changed in 1831, it remained expensive and never really formed a prominent part of British foodways (Gordon 2015). Upon arrival in Upper Canada in the first half of the 19th century, all previous restrictions on venison were gone. It makes sense then that many personal letters written to family and friends in England would highlight sudden access to a previously forbidden food. When reading these letters, one is struck by their positive tone regarding life in Upper Canada: the abundance of food, the ease with which they settled on the land, and their general ability to survive. Few make allusions to the difficulties involved with living in a frontier environment and establishing a new farm. These letters can be seen as reassurances to loved ones about their new lives and the choices they made to immigrate to Canada, as opposed to accurate accounts of daily lives. It is also worth noting that authors like Traill, Moodie, and Langton, who extolled the abundance of venison, represent members of the upper-middle class and were likely more open to the consumption of wild game, as Gordon (2015) suggests. Their experiences of Upper Canada, upon which many historical summaries are based, do not necessarily reflect those of most other Upper Canadian farmers. Archaeological excavations of the Moodie household revealed the largest deer assemblage of any site investigated here, suggesting venison played an important role in that household’s diet.

Other mammalian species, such as squirrels, muskrats, beaver, bear, lynx, fox, porcupine, and woodchuck, are occasionally recorded as being consumed, but these are found irregularly (and only in small numbers) in the archaeological record. Wild hare, abundant in the forests of Upper Canada, are quite similar to European hare (*Lepus europaeus*) and domestic rabbits (*Oryctolagus cuniculus*). These could be caught in the same manner, and recipes suggest they were incorporated into the diet in comparable ways, although Traill (1857:155) suggests the taste was slightly inferior to European varieties. New duck species were available in Canada, but personal correspondence again suggests people just thought of these as ducks, hunting and eating them as they would back in Britain. Wild turkeys were introduced to Britain in the later medieval period and were a familiar part of British foodways by the 18th and 19th centuries (Fothergill 2014). Wild geese, historically described as “fishy and oily” (Traill 1857:156), differed significantly from the domestic greylag goose, and evidence suggests they were not often consumed in Upper Canada, where plenty of greylag remains were identified instead. Passenger pigeons (*Ectopistes migratorius*) looked and behaved differently from the common rock pigeon, but were, by all accounts, very easy to catch and, at one point, very abundant, passing through Upper Canada in flocks of billions in the spring and autumn (Simcoe 1965:111; Greenberg 2014:91–96). However, they never featured heavily in local foodways. A less-than-enthusiastic incorporation of passenger pigeon into the Upper Canadian diet is of little surprise, as some historical texts describe their questionable flavor (Greenberg 2014:71), but this did not stop residents from killing the birds for sport. Mitchell (1935:120) describes how the yearly arrival of the passenger pigeons in early 19th-century Toronto made the city sound like a war zone, as people could not resist firing at such easy targets.

Freshwater fish represent the only local fauna exploited more than domestic or imported species of the same taxonomic class. While fish represent a relatively small portion of archaeological assemblages (emphasized as a result of biasing recovery strategies), a variety of local species are identified in the assemblages, and few imported species appear to be incorporated into diets. The proportion of fish in the overall assemblages is in keeping with contemporaneous archaeological materials recovered in England and the United States (Landon 1997; Kuehn 2007; Scott 2007; Gordon 2015; Heinrich and Giordano 2015). The lack of popular marine species, such as cod (*Gadus morhua*) and haddock (*Melanogrammus aeglefinus*), likely relates to the inability of marine fish to remain fresh while being transported to this province (Tourigny 2016). Only assemblages located along major shipping routes contained evidence of imported marine species. The most popular Ontario species described in the documents were salmon or trout, muskellunge, whitefish, and black bass. Other species, such as perch, sunfish, rock bass, and freshwater eel, are also described as occasionally present at the dinner table (Brown 1849:83; Traill 1857:162; Langton 1926:34–35). The fish in some places were apparently so plentiful that “in some parts of the lake, if you are short of meat for dinner, you may put the potatoes on to boil and before they are done enough, you may have ten or twenty bass on the grid-iron” (Langton 1926:34–35). While immigrants from the northeastern United States would have been familiar with Ontario species, species closely related to Ontario pike, trout, eel, and salmon were also available in the UK. Most documents simply refer to fish as fish and not the individual species they represented.

The similarity in fauna between Upper Canada and Britain leaves very few local species unfamiliar to British immigrants and not already included in previous foodways. Bear and moose are two large land mammals that have no counterparts in Britain, and these never formed a part of the Upper Canadian diet, despite their edibility. Their unfamiliarity to the British palate is one possible reason for their rejection, as these types of meat are today known for their “gaminess.” Such an explanation may also justify the exclusion of deer and passenger pigeons from the Upper Canadian diet. Undesirable taste and flavor was once mentioned as a reason people avoided eating Canada geese and passenger pigeons (Traill 1857:156; Greenberg 2014:72), and it is entirely possible that Canadian deer, feeding off a landscape significantly different from the managed British countryside, carried a gamier flavor. The gaminess and dryness of the meat would have required different, possibly unfamiliar, or less desirable cooking and storage practices. It is also possible that deer were not a reliable meat source where hunting took time away from agricultural duties, and markets were not regularly provisioned with the product. Commercially viable techniques for long-term storage, transportation, and provision of venison for markets were not developed, leaving the majority of residents to choose farm-sourced meat options.

## Development of Upper Canadian Traditions?

While the foodways of early British and American settlers of Upper Canada can be described as a continuation of previously held foodways, did these eventually lead to foodways that became characteristic of the region? Similarities in food-consumption patterns with their southern neighbors are of little surprise, as a large number of Americans settled in the province and the border was readily crossed by people and goods. However, the water border with the United States and differences in political views could have acted as a “dividing screen,” allowing Upper Canadians to further associate with their British roots (Careless 1984:11). Similarly, the new environment in which the British immigrants suddenly found themselves would have had an effect on the foods they consumed. The occasional lack of fresh meat, the emphasis on barreled meat products, different preparation techniques, and the incorporation of indigenous flora led to what can be described as a different cuisine in Upper Canada (Traill 1846, 1857; Moodie 1853). Paleobotanical research has identified the use of native North American fruits and vegetables in the diets of soldiers stationed at Fort Wellington in Prescott, Ontario (Moyle 1994; Lyall 2010), but the extent to which wild flora were incorporated into domestic foodways is unclear.

Earlier scholarship suggested Upper Canadian diets relied heavily on pork and potatoes. While the European Canadian and American Loyalist assemblages identified here may at first appear to be homogenous, with a focus on domesticates and a near avoidance of wild resources, closer inspection shows each one to be unique. Some assemblages relied heavily on pork, while others relied on beef, and a few others ate more mutton than their neighbors. The majority of farmers continued to focus on raising cattle and pigs over the course of the 19th century, and sheep do not play an increasingly greater role in local diets, contrary to previous suggestions (Need 1838:90; Ferris and I. Kenyon 1983). Access to preserved meat products through local trade and markets was almost universal in Upper Canada during the 19th century, indicating most households had access to farm-sourced meat throughout the year. While access to fresh seafood was limited by one’s proximity to urban markets or port towns, general meat-consumption patterns could not be related to urban vs. rural or temporal divides. Therefore, the diversity observed among the deposits boils down to individual preferences and/or circumstances.

Preserved meat in the form of barreled products formed an important part of Upper Canadian foodways (Tourigny 2017a). Beef was raised locally in Upper Canada and likely consumed fresh, while pork was either local or imported (Guiry et al. 2017; Tourigny 2017a). Imported pork barrels were more common in the urban markets of Toronto, while local pork-barreling activities were identified in Upper Canada’s rural regions (Guiry et al. 2017; Tourigny 2017a). Rural areas likely consumed locally prepared pork barrels, and the city of Toronto eventually became a renowned pork-packing center itself in the late 19th century (Kheraj 2013:135). Early accounts suggest residents in the backwoods would travel great distances to procure enough preserved pork to last the winter (Langton 1926:57) and so were not necessarily reliant on fresh venison. While Upper Canadian food traditions might not be as dependent on wild resources as originally suspected, they did come to be characterized by the consumption of both pork and beef. The style in which these meats were prepared and cooked may have been influenced by the local availability of fresh produce and, perhaps more importantly, the unique issues surrounding the preparation of barreled meat products. Tourigny (2017a) notes barreled pork was prone to preservation issues: the meat often went bad, and determined cooks, unwilling to let items go to waste, would need to cook away the rancidity and salt. Many dishes credited as popular in the province consist of meats prepared in a way that masks flavors and/or textures amongst other ingredients (e.g., soups and stews); U. Abrahamson (1967) and Bates (1978) summarize common Upper Canadian recipes. A reliance on greasy, salty, and occasionally rancid supplies of barreled meat may be seen as one of the earliest shared experiences that helped form Upper Canadian foodways and identities (Tourigny 2017a).

With the arrival of the railways in the 1850s, improved preservation technology (refrigeration and tinning), and expanding markets, Upper Canadian foodways became increasingly dependent on market resources and international commercial links. Although evidence of a “frontier effect”' (Scott 1985, 1991, 1996) was still somewhat evident in some remote locations in the early 19th century, the scarcity of archaeological deposits dating to this time period in Upper Canada and the rapid development of a market economy likely obliterated most evidence of this in the province.

## Maintaining Traditions—Food as a Reflection of Success

Recalling the importance of food as central to the formation of individual and group identities, the results presented here speak to the importance of maintaining traditional foodways amongst immigrant populations in Upper Canada. The quotidian nature of food consumption and preparation, and the symbolic acts often associated with these, make foodways a useful way to investigate the ways groups maintained solidarity and identity (Wilk 1999; Weller and Turkon 2015) and how immigrants can draw on foodways to further strengthen cultural identities when faced with change (Ferrero 2002; Cook 2008).

While immigrants to Upper Canada inevitably faced some change in foodways upon arrival (Norman 2012), historical records suggest that those who could afford it made efforts to maintain the foodways to which they were accustomed (Roberts 2012). This research demonstrates the extent to which most immigrants also tried to maintain food traditions. Anthropologists argue that, among immigrants, foodways play a central role by reminding them of former lives and shaping their identities as they build their futures (Mares 2012). This article similarly argues that previously held foodways were important to British and Loyalist immigrants in Upper Canada. In addition to using foodways as a reminder of past lives and as a symbol of group identity, I believe maintenance of traditional foodways was also seen as reflection of a successful life in their new country. Upon arrival to the Upper Canadian countryside, new settlers were tasked with clearing their land, building access roads, and establishing themselves as farmers. The Upper Canadian economy grew through the production and exportation of wheat in the first half of the 19th century (R. Jones 1946). If rural residents sought to live a life surrounded by the hallmarks of a successful farm, then subsisting primarily on livestock, grains, and cereals could be seen as evidence of success. The source of domestic meat, whether raised on one’s own farm or purchased elsewhere, did not necessarily matter, as long as familiar foods were consumed at the table. If the majority of farms were concerned with crops over livestock, it is not surprising that many continued to rely on the import of barreled products to supply meat to the table. Similarly, if Upper Canada’s urbanites were to emulate life in other successful cities, a focus on commercially sourced livestock was crucial. Early settlers would have been proud of their accomplishments (Duncan 2012:92), and the following passage from a letter Elizabeth Russell wrote to a friend in England in 1793 highlights an emphasis on recreating familiar foodways:

We are comfortably settled in our New House and have a nice little farm about us. We eat our own Mutton and Pork and Poultry. Last year we grew our own Buck wheat and Indian corn and had two Oxen, got two cows with their calves with plenty of pigs and a mare and Sheep. We have not made butter yet but hope soon to do so. (Duncan 2012:92)

## Conclusion

Early Upper Canadian foodways were heavily influenced by the British and American working-class traditions from which they came. Immigrants to the province wanted to create a sense of normalcy in their everyday routines by incorporating the foods they were accustomed to eating prior to their arrival in the region. It is evident that meat from domestic animals was relied upon from the initial moments of settlement, while wild sources of meat were only occasionally incorporated. With the exception of marine resources, the distance from urban centers was not a determining factor for accessing either wild or domestic sources of meat. Pork played an important role for many families; however, the consumption of beef was equally important, if not more important, to other households. A cuisine or tradition characteristic of the region eventually developed, in large part as a result of cooking with barreled meat products and the likely incorporation of local flora. Further research into the extent to which wild flora were incorporated into diets would provide a more complete picture of the development of Upper Canadian foodways.
